# Making healthcare accessible for single adults with complex needs experiencing long-term homelessness: A realist evaluation protocol

**DOI:** 10.12688/hrbopenres.13154.2

**Published:** 2021-01-21

**Authors:** Rikke Siersbaek, John Ford, Clíona Ní Cheallaigh, Sara Burke, Steve Thomas

**Affiliations:** 1 Centre for Health Policy and Management, Trinity College Dublin, Dublin 2, Dublin, Ireland; 2 Institute of Public Health, University of Cambridge School of Clinical Medicine, Cambridge, CB2 0SR, UK; 3 Department of Clinical Medicine, Trinity College Dublin, Dublin 8, Dublin, D08 NHY1, Ireland

**Keywords:** Homelessness, Homeless adult, Inclusion health, Access to healthcare, Realist evaluation

## Abstract

**Background:** Over the last several years, homelessness has increased in Ireland and across Europe. Rates have recently declined since the coronavirus disease 2019 (COVID-19) pandemic, but it is unclear whether emergency housing measures will remain in place permanently. Populations experiencing long-term homelessness face a higher burden of multi-morbidity at an earlier age than housed populations and have poorer health outcomes. However, this population also has more difficulty accessing appropriate health services. A realist review by the authors found that important health system contexts which impact access are resourcing, training, funding cycles, health system fragmentation, health system goals, how care is organised, culture, leadership and flexibility of care delivery. Using a realist evaluation approach, this research will explore and refine key system-level factors, highlighted in our realist review, in a local health care system.

**Aim: **The aim of this study is to understand how funding procedures and health system performance management impact service settings, staff, providers and their ability to make services accessible to populations experiencing homelessness.

**Methods**: A realist evaluation will be undertaken to explain how funding and health system performance management impact healthcare accessibility for populations experiencing homelessness. Data will be collected using qualitative and realist interview techniques and focus group methodology. Secondary data such as policy documents and budgets will utilised. The analysis will follow Pawson and Tilley’s iterative phases starting with building an Initial programme theory, then data collection, data analysis, synthesis and finally building a refined programme theory.

**Conclusion**: Building on a realist review conducted by the same research team, this study will further test and refine findings that explain how health system factors impact healthcare accessibility for populations experiencing homelessness. The study has the potential to inform policy makers, health planners and managers of contextual factors that can be modified to increase healthcare accessibility.

## Introduction

Homelessness has been on the rise in Ireland and internationally over the last several years. From March 2015 to March 2020, the total number of people experiencing homelessness in the Republic of Ireland (including single adults and families with children) grew from a total of 4,261 people to 9,335 representing an increase of 119 percent ^1^. The total number of people being reported as homeless has declined since the outbreak of coronavirus disease 2019 (COVID-19) to 8,728 people in July 2020 ^1^. However, it is unclear whether emergency housing measures will be in place permanently ^2^.

According to the European Typology of Homelessness and Housing Exclusion (ETHOS) homelessness occurs on a continuum including the following experiences:

- Rooflessness (sleeping rough, without any shelter)
- Houselessness (having somewhere to sleep but temporary in shelter or institution)
- Living in insecure housing (insecure tenancies, threat of eviction, violence)
- Living in inadequate housing (overcrowding, unfit housing, caravans on illegal campsites) ^3^

The homelessness statistics in Ireland cover several different populations with somewhat differing needs, including single adults and families. The people included in the statistics are those relying on emergency accommodation and does not include people who are staying with family members, rough sleeping, camping, etc ^1, 4^.

In addition to the physical aspects of homelessness there is an important temporal aspect. Researchers use three temporal categories: crisis homelessness, intermittent homelessness or chronic homelessness. Crisis homelessness occurs for people who experience sudden homelessness after an eviction, divorce or job loss. Intermittent homelessness occurs for people who move in and out of homelessness interrupted by access to adequate housing or institutional care stays. Chronic homelessness is defined as experiencing homelessness lasting more than a year continuously or for individuals with a disabling condition, four episodes of homelessness over a period of two years ^5^.

The focus of this research is on the subset of the homeless populations who are single adults experiencing chronic homelessness. This population group experiences lengthy periods of homelessness while moving between any or all types of accommodation outlined in the ETHOS typology, sometimes also with time spent in institutional settings and/or in sufficient accommodation ^5^. Such people typically have lived lives marked by trauma from a young age and often have complex health and social care needs ^6, 7^. Many experience tri-morbidity, that is the simultaneous presence of physical and mental ill health and substance use disorders ^8^. As a result, populations who experience homelessness and other forms of social exclusion have higher levels of morbidity than other populations and the onset of morbidity and multimorbidity happens at an earlier age ^9– 11^. Homeless people also tend to die at a much younger age than their housed peers ^10, 11^. In Dublin the median age of death for homeless people who die while accessing Dublin Region Homeless Executive supported services is 42 years old (44 years old for males and 37 years old for females) ^12^. In line with Tudor Hart’s inverse care law, this high-need population generally has more difficulty accessing healthcare than other populations, despite their poorer health and low life-expectancy ^7, 13– 16^.

The majority of research examining healthcare access for populations experiencing homelessness is focused on the patient journey from the perspective of the individual seeking to access health services ^13– 15, 17– 21^. While it is important to understand the factors that make healthcare accessible for highly vulnerable population groups, there is a risk that this focus leads to inappropriately placing responsibility for accessing healthcare with populations experiencing homelessness and not with the health system. To remedy that imbalance, this research aims to understand access from a systems perspective.

Health systems are also characterised by a high level of complexity as they consist of a number interacting people, places, policies and actions as described in the WHO’s definition of a ‘health system’: ‘(i) all the activities whose primary purpose is to promote, restore and/or maintain health; (ii) the people, institutions and resources, arranged together in accordance with established policies, to improve the health of the population they serve, while responding to people’s legitimate expectations and protecting them against the cost of ill-health through a variety of activities whose primary intent is to improve health’ ^22^. In other words, a health system is an open system with many parts, at times interacting, and moving along non-linear pathways on diverse timelines. At the intersection with patients experiencing homelessness seeking to access healthcare services, health systems contribute to a number of intended and unintended outcomes which emerge as more than the sum of their parts (eg a patient may nominally access healthcare in the emergency department but leave before being seen even through physical access is possible due to a stigmatising culture which makes the environment unwelcoming) ^23– 27^

Realist approaches (realist review and realist evaluation) are designed for understanding complex phenomena. The realist logic of inquiry is based on an understanding of reality as existing independent of our ability to observe it. It views the world as being one where we cannot observe or measure many of the processes that produce outcomes we are interested in ^28^. For example, in seeking healthcare access, a person experiencing homelessness may be met with stigma and feel unwelcomed and as a result decide not to seek care. In this case, we can measure the lack of realised healthcare access in low healthcare utilisation. However, we cannot independently observe and measure stigmatising attitudes nor can we measure the response arising in the person experiencing stigma. In the realist view of the world, we can theorise about what is happening to cause the outcome of poor healthcare access by understanding the social and psychological processes commonly at play in situations where health services prove accessible or inaccessible for populations experiencing homelessness.

In the school of Pawson and Tilley ^29, 30^, realist evaluation seeks to understand the underlying mechanisms that generate a given outcome in a particular context using primary data. It is methods neutral and can employ data sources from a variety of study designs and methods. Realist approaches seek to uncover the conditions in which something works and for whom it works and understanding why rather than merely whether something works or not.

**Figure 1.**
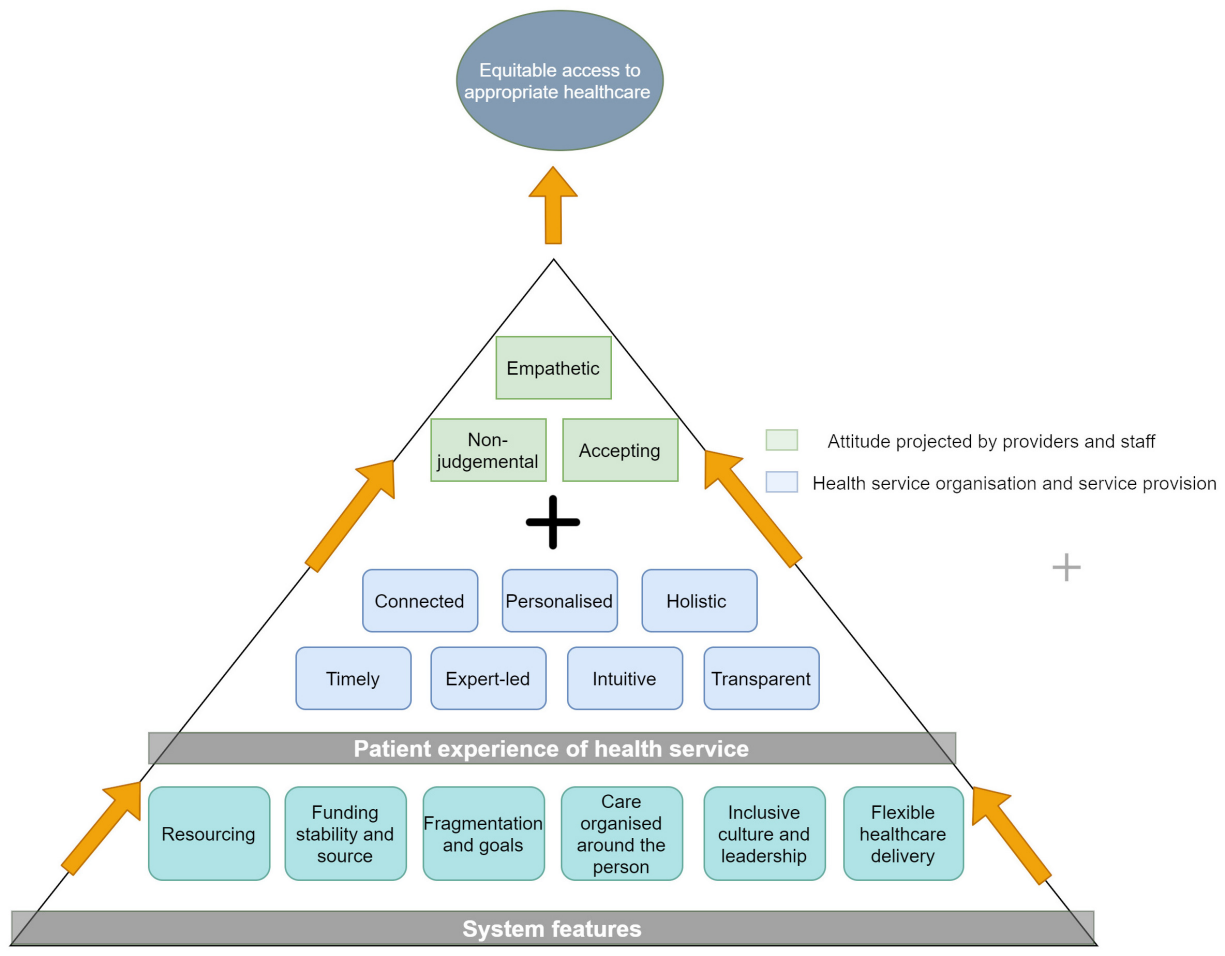
Full programme theory of health system features that contribute to healthcare accessibility for homeless populations ^31^.

To add to the understanding of health system factors that improve access to healthcare for long-term homeless adults, we carried out a realist review ^31^. This realist review produced a number of context-mechanism-outcome configurations (CMOCs) and an overarching programme theory (see Figure 1). This programme theory synthesised the full set of findings from the review showing the interlinking set of factors which must all be in place for health systems to successfully provide healthcare access to populations experiencing homelessness. The programme theory explains that important health system contexts which impact access are resourcing, training, funding cycles, health system fragmentation, health system goals, how care is organised, culture, leadership and flexibility of care delivery. Key mechanisms which arise in these contexts are provider attitudes, provider confidence, staff and provider experience of stability and sustainability, organisation-centred thinking, flexibility, transparency, timeliness, demonstration of respect and empathy, trust, adaptability, and anticipation.

Conversely, when those system features are partially in place or not in place at all, the patient experience of health services is impacted negatively. Eg when health services are fragmented, care cannot be delivered in a timely manner because each service or speciality operates on their own schedule and each step on a patient pathway depends on a referral from the last.

This study will focus on a subset of the findings of our realist review to evaluate how funding procedures and health system performance management impact service settings, staff, providers and their ability to make themselves accessible to populations experiencing homelessness. We have chosen to further investigate these two areas of study because they are under-researched and they will contribute to the work of planners and policy makers ^32– 34^.

## Study design

In this study, we will use realist evaluation, a theory-driven approach well-suited for analysing complex topics and interventions as detailed above.

Data collection will take place in Dublin with the goal of learning lessons particular to how health services make themselves accessible or not to populations experiencing homelessness. The study will be limited to Dublin region homeless healthcare services because the majority of homeless adults in Ireland live in Dublin ^35^ and the majority of healthcare services are provided in Dublin ^36^. Transferrable findings that will be useful internationally will be generated.

The study will employ documentary analysis of policy documents (Health Service Executive (HSE), Department of Health, non-government organisation (NGO) sector) and health service utilisation and budgeting statistics, as well as semi-structured and realist interviews with stakeholders in the health and NGO sectors. This study will also include focus groups with individuals with lived experience of homelessness who will aid in challenging, confirming and further refining study findings. 

**Figure 2.**
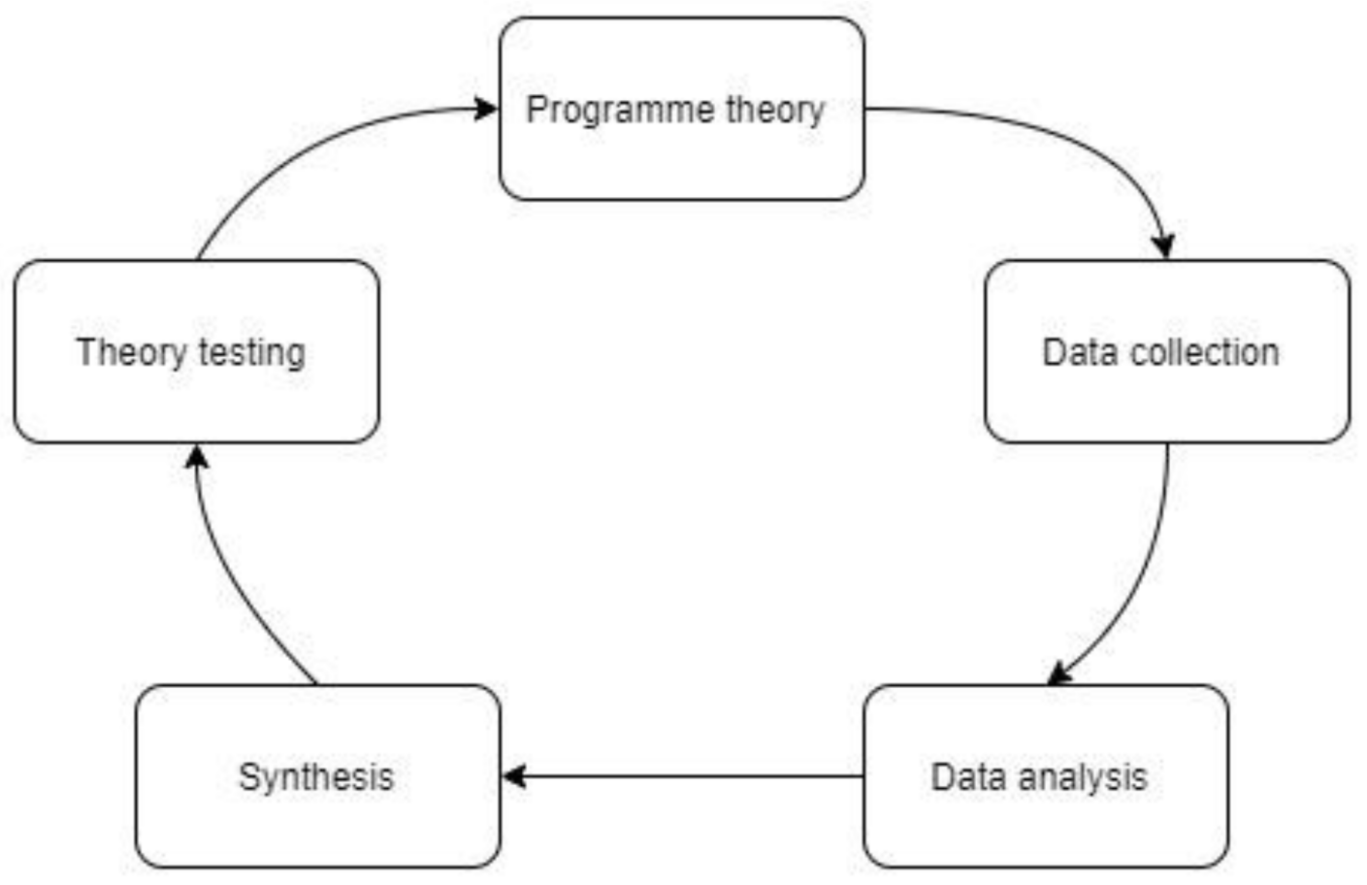
Stages of a realist evaluation.

The study will follow the iterative realist evaluation design as set out by Pawson and Tilley ^30^ in the following phases ( Figure 2):

1. Initial programme theory building
2. Data collection
3. Data analysis
4. Synthesis
5. Refined programme theory building

The phases will repeat as needed. If it becomes clear that more data or different sources are needed, these will be incorporated into the synthesis in order to produce the most complete refined programme theory possible.

### Initial programme theory

Theory building is the start and the end of realist work – the work starts with an initial programme theory and ends with a refined one after iterative rounds of data gathering and theory building.

Building on our realist review, the team has selected two context-mechanism-outcome configurations (CMOCs) to further explore and refine. The review indicates what is known about what works in promoting healthcare access in homeless populations from a health systems perspective – see the full programme theory above in Figure 1. Due to time and resource constraints it is not possible to further test the full set of six detailed CMOCs generated in the review.

For this study we will focus on two particular areas of the full programme theory explained in the two CMOCs described below. These relate to:

- How health services are funded
- How health systems manage performance

We have chosen to further investigate these two areas of study because they are under-researched and they will contribute to the work of planners and policy makers ^32– 34^.

Building on the below CMOCs from our review, we will, where possible, further explain and add to the relationships between the contexts, mechanisms and outcomes identified in the international literature by expanding the analysis with the use of primary data and additional secondary data sources.

**Figure 3.**
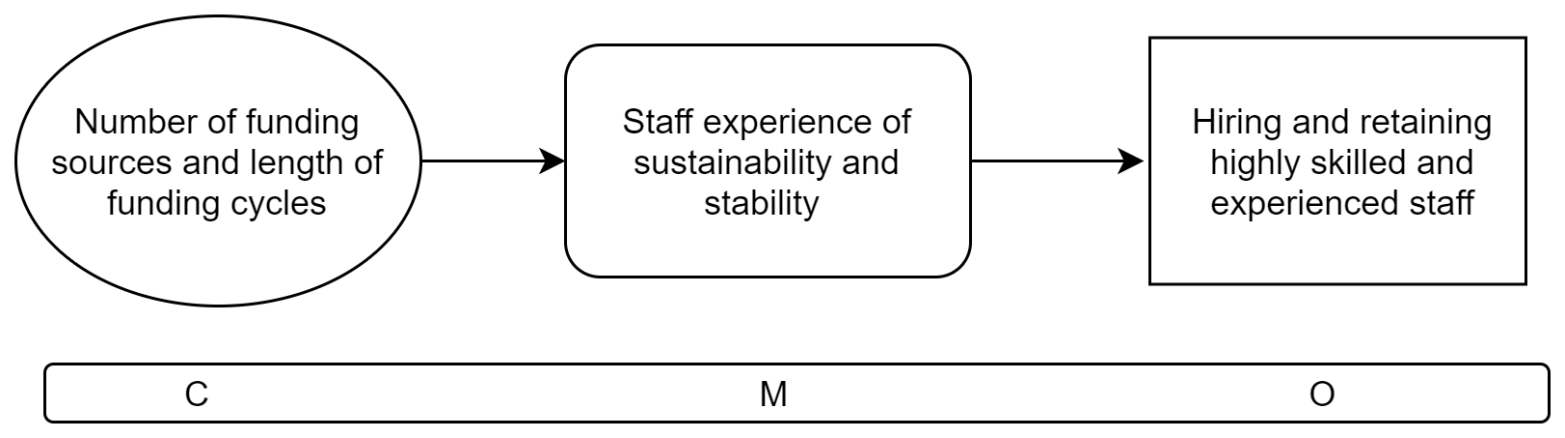
CMOC 1: Funding stability.

CMOC1 ( Figure 3) suggests that when funding cycles are short, unreliable and come from multiple sources, eg grant funding to meet a specific need ^34, 37– 39^, services lack sustainability and stability ^1, 34, 40– 42^ and as a result face difficulties hiring and retaining skilled and experienced staff members ^34, 40– 43^.

**Figure 4.**
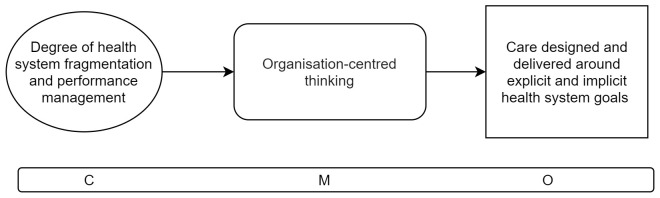
CMOC 2 - Health system fragmentation and performance management.

CMOC2 ( Figure 4) suggests that when the parts of a health system operate in silos with narrowly defined goals ^34, 38, 39, 44, 45^, performance management is aligned with meeting those goals and they become the priority of staff even if they are not aligned with the needs of their patients ^39, 44, 45^. Performance management practices have tended to inhibit the ability of staff to deliver services in holistic, coordinated and flexible ways. As a result, healthcare is organised around the needs of providers and the system not the person ^15, 38, 39, 44– 46^.

Building on these CMOCs and the understanding of the underlying causative relationships they describe, the analysis will evaluate how funding procedures and health system performance management impact service settings, staff, providers and their ability to make themselves accessible to populations experiencing homelessness.

We will seek to answer the following questions:

- How are health services for homeless populations funded?
- For whom do funding arrangements work and not work, and why?
- How is health system performance management intended to translate into service design and delivery for populations experiencing homelessness?
- In what circumstances are health system performance management and organisational targets useful for creating healthcare accessibility for homeless populations and when are they not?
- Who (patients, staff, providers, the system) does health system performance management work for, to what extent?

### Data collection

Data will include grey literature and secondary sources identified through internet searching and snowballing as well as primary data collected via qualitative and realist interviews and focus groups. Realist interview/ focus group guides are available as extended data ^47^. Approximately ten key informant interviews will take place with professionals with significant experience in preventive, primary and secondary healthcare sectors and NGOs that provide services to homeless, and the HSE National Office for Social Inclusion, as well as relevant experts and academics. These will be identified by members of the research team which has broad networks in health and social services for homeless populations in Ireland. Snowball sampling will also be used to identify further interviewees. Interviews will take place via online video conferencing due to the current COVID-19 pandemic or in person, if and when possible. Interviews will be recorded and transcribed. 

The research team will also conduct at least one focus group with experts by experience (individuals with lived experience of homelessness). An agreement to engage with individuals who are part of the Depaul peer health worker panel is in place but it remains to be seen if COVID-19 restrictions will allow us to proceed. It is likely that several focus groups conducted with subgroups within the target population would be useful. Women who have experienced homelessness may feel more comfortable participating in a women-only group and the same may be the case for LGBT or migrant people. If possible, given time and COVID-19 constraints, the research team intends to conduct more than one focus group. If holding focus groups is not possible, other methods will be considered. The goal of the focus groups is to add a service user’s perspective to the analysis of factors that impact health accessibility, particularly uncovering ones which may not be evident to individuals who have not personally had the experience of homelessness.

Data collection will be iterative and additional data sources may be collected as needed after the first data analysis phase to substantiate findings from interviews and focus groups. Eg if a particular policy or budget or procedure is discussed by a source, the research team will endeavour to access documentation regarding the item being discussed to add it to the analysis beyond the interviewee’s opinion.

### Data analysis

All collected data will be transcribed and then inputted and analysed in NVivo software. For key informant interviews, a set of initial codes will be generated inductively while coding the first several interviews. The same codes will then be used across the rest of the interviews with more added as needed. Data collected from the focus group will likely have some similar codes and some different codes due to the difference in perspective and will be coded separately and inductively. Policy documents and grey literature sources will also be included in the analysis and will be coded separately from the primary data with a set of initial codes being developed inductively from what the team deem to be the likely most useful sources. Subsequent documents will be coded deductively using those codes while adding more as needed. Sources which may be identified over the course of the data collection from interviews and focus groups will be coded using the same procedure.

### Synthesis

Each set of data (interviews, focus groups and secondary literature) will be analysed separately and will then be synthesised at the interpretation stage using triangulation ^48^ to formally explore the agreement and disagreement between sources and to identify how these contribute to the analysis. The goal is not to only find instances where there is agreement between sources but also to investigate areas of disagreement or where findings only occur in one set of sources. Eg service users may have a perspective on healthcare accessibility that is invisible to planners and providers of services but which is nonetheless important to understanding how services are and are not made accessible. 

Following the data triangulation, CMOCs will be built using a combination of data sources. CMOCs will be iteratively refined and further developed as needed based on team discussions. A final programme theory will be constructed from CMOCs at the end of the project.

### Ethics

Ethical approval has been granted by the Trinity College Dublin Health Policy and Management and Centre for Global Health Research Ethics Committee (application 22/2019/01).

Given that the population in question is a highly vulnerable one, the team is keenly aware of our responsibility in conducting this research ethically and sensitively. Trusted and known gatekeepers will be used to recruit focus group participants to provide every opportunity for potential participants to say no to participating for any reason. Participants will also be given a thorough oral explanation of the informed consent form along with the written copy of the document to ensure that they understand that participation is voluntary and that they may leave the study at any time. A professional who is known to the individuals in the peer health worker panel and who has significant experience working with the population, will take part in moderating the focus group in order to help provide a familiar and safe environment. Should a need for any support services arise as a result of participation in the focus group, Dr Cliona Ni Cheallaigh, a member of the research team and the clinical lead for the Inclusion Health Team in St. James’s Hospital Dublin will personally ensure that the individual or individuals are given the right care and treatment.

### Dissemination of information

The main product of this research will be a research article which will be disseminated via a peer-reviewed journal. It will also be disseminated to research, policy making and practice networks, and through the Centre for Health Policy and Management, Trinity College Dublin website.

### Study status

Documentary analysis and data collection via interviews has commenced. Three pilot interviews have been completed. Realist interviews are underway. Data analysis and synthesis is yet to be done.

## Discussion

To our knowledge this is the first realist evaluation seeking to explain how funding procedures and health system performance management impact service settings, staff, providers and their ability to make themselves accessible to populations experiencing homelessness.

With the predominance in the literature of studies examining individual level factors that impact on healthcare access, there is a lack of understanding of the impact of system level decisions and priorities on downstream health service delivery for socially excluded populations. This study will add important knowledge to the field by specifically examining health system contexts and mechanisms.

A strength of this study is the use of a realist approach. The study will uncover modifiable contexts which impact upon healthcare accessibility and will be of interest to policy makers and healthcare planners and managers. A potential impact of the study is to inform health system and policy leaders, with the ultimate aim of making healthcare more accessible for populations experiencing homelessness.

## Data availability

### Underlying data

No underlying data are associated with this article

### Extended data

Open Science Framework: Making healthcare accessible for single adults with complex needs experiencing long-term homelessness: A realist evaluation protocol. https://doi.org/10.17605/OSF.IO/GCHFD ^47^

This project contains the following extended data:

- Realist interview-FG guide_Rikke Siersbaek.docx (Interview and focus group guides)

Extended data are available under the terms of the Creative Commons Attribution 4.0 International license (CC-BY 4.0).

## References

[1] Focus Ireland: Latest Figures on Homelessness in Ireland.2020 Reference Source

[2] Focus Ireland: Homeless numbers drop for a fifth consecutive month to 8,699 as Focus Ireland calls on government to keep vulnerable renters safe in their homes.Focus Ireland.2020 [cited 2020 Aug 25] Reference Source

[3] FEANTSA: What is ETHOS ? European Typology of Homelessness.2017;13–13. Reference Source

[4] Long AE Sheridan S Gambi L: Family Homelessness in Dublin: Causes, Housing Histories, and Finding a Home Full Report.Focus Ireland; (Insights into family homelessness). Report No.: 2019;2(1):72 Reference Source

[5] Fazel S Geddes JR Kushel M: The health of homeless people in high-income countries: descriptive epidemiology, health consequences, and clinical and policy recommendations. *Lancet.* 2014;384(9953):1529–40. 10.1016/S0140-6736(14)61132-6 25390578 PMC4520328

[6] Hopper EK Bassuk EL Olivet J: Shelter from the Storm: Trauma-Informed Care in Homelessness Services Settings. *Open Health Serv Policy J.* 2010;3:80–100. Reference Source

[7] Luchenski S Maguire N Aldridge RW: What works in inclusion health: overview of effective interventions for marginalised and excluded populations. *Lancet.* 2018;391(10117):266–80. 10.1016/S0140-6736(17)31959-1 29137868

[8] Cornes M Whiteford M Manthorpe J: Improving hospital discharge arrangements for people who are homeless: A realist synthesis of the intermediate care literature. *Health Soc Care Community.* 2018;26(3):e345–59. 10.1111/hsc.12474 28730744

[9] de Sousa E O’Gara R Power C: Premature Ageing in the Homeless Population.Dublin: Depaul;2018 Reference Source

[10] O’Reilly F Barror S Hannigan A: Homelessness: An unhealthy State Health Status, Risk Behaviours and Service Utilisation among Homeless People in Two Irish Cities. *Partnership for Health Equity.* 2015;97 Reference Source

[11] Queen AB Lowrie R Richardson J: Multimorbidity, disadvantage, and patient engagement within a specialist homeless health service in the UK: an in-depth study of general practice data. *BJGP Open.* 2017;1(3):bjgpopen17X100941. 10.3399/bjgpopen17X100941 30564673 PMC6262212

[12] Ivers JH Barry J: Mortality amongst the homeless populatin in Dublin.Dublin: Department of Public Health and Primary Care. Institute of Population Health, School of Medicine, Trinity College Dublin;2018 Reference Source

[13] Campbell DJT O’Neill BG Gibson K: Primary healthcare needs and barriers to care among Calgary’s homeless populations. *BMC Fam Pract.* 2015;16(1):139. 10.1186/s12875-015-0361-3 26463577 PMC4603688

[14] O’Carroll A Wainwright D: Making sense of street chaos: an ethnographic exploration of homeless people’s health service utilization. *Int J Equity Health.* 2019;18(1):113. 10.1186/s12939-019-1002-6 31337407 PMC6651952

[15] O’Donnell P Tierney E O’Carroll A: Exploring levers and barriers to accessing primary care for marginalised groups and identifying their priorities for primary care provision: a participatory learning and action research study. *Int J Equity Health.* 2016;15(1):197. 10.1186/s12939-016-0487-5 27912783 PMC5135741

[16] Hart JT: THE INVERSE CARE LAW. *Lancet.* 1971;1(7696):405–12. 10.1016/s0140-6736(71)92410-x 4100731

[17] Argintaru N Chambers C Gogosis E: A cross-sectional observational study of unmet health needs among homeless and vulnerably housed adults in three Canadian cities. *BMC Public Health.* 2013;13(1):577. 10.1186/1471-2458-13-577 23764199 PMC3691921

[18] Baggett TP O’Connell JJ: The Unmet Health Care Needs of Homeless Adults: A National Study. *Am J Public Health.* 2010;100(7):1326–33. 10.2105/AJPH.2009.180109 20466953 PMC2882397

[19] Booth ML Bernard D Quine S: Access to health care among Australian adolescents young people’s perspectives and their sociodemographic distribution. *J Adolesc Health.* 2004;34(1):97–103. 10.1016/j.jadohealth.2003.06.011 14706412

[20] Kertesz SG McNeil W Cash JJ: Unmet Need for Medical Care and Safety Net Accessibility among Birmingham’s Homeless. *J Urban Health.* 2014;91(1):33–45. 10.1007/s11524-013-9801-3 23620012 PMC3907626

[21] Salem BE Nyamathi A Idemundia F: At a Crossroads: Reentry Challenges and Healthcare Needs Among Homeless Female Ex-Offenders. *J Forensic Nurs.* 2013;9(1):14–22. 10.1097/jfn.0b013e31827a1e9d 24078800 PMC3783031

[22] WHO: Health Systems Strengthening Glossary.WHO. World Health Organization; [cited 2020 Nov 17]. Reference Source

[23] Aday LA Andersen R: A Framework for the Study of Access to Medical Care. *Health Serv Res.* 1974;9(3):208–220. 4436074 PMC1071804

[24] Davidson PL Andersen RM Wyn R: A Framework for Evaluating Safety-Net and other Community-Level Factors on Access for Low-Income Populations. *Inquiry.* 2004;41(1):21–38. 10.5034/inquiryjrnl_41.1.21 15224958

[25] Dixon-Woods M Cavers D Agarwal S: Conducting a critical interpretive synthesis of the literature on access to healthcare by vulnerable groups. *BMC Med Res Methodol.* 2006;6:35. 10.1186/1471-2288-6-35 16872487 PMC1559637

[26] Evans DB Hsu J Boerma T: Universal health coverage and universal access. *Bull World Health Organ.* 2013;91(8):546–546A. 10.2471/BLT.13.125450 23940398 PMC3738317

[27] Levesque J-F, Harris MF Russell G: Patient-centred access to health care: conceptualising access at the interface of health systems and populations. *Int J Equity Health.* 2013;12(1):18. 10.1186/1475-9276-12-18 23496984 PMC3610159

[28] Jagosh J: Retroductive theorizing in Pawson and Tilley’s applied scientific realism. *J Crit Realism.* 2020;19(2):121–30. 10.1080/14767430.2020.1723301

[29] Pawson R: Evidence-based policy: a realist perspective.London ; Thousand Oaks, Calif: SAGE;2006;196 Reference Source

[30] Pawson R Tilley N: Realistic Evaluation. Sage Publications Inc;1997 Reference Source

[31] Siersbaek R Ford J Burke S: Contexts and mechanisms that promote access to healthcare for populations experiencing homelessness: a realist review. *BMJ Open.*Under review. 10.21203/rs.3.rs-79236/v1 PMC10583475 37848878

[32] Crane M Cetrano G Joly L: Mapping of specialist primary health care services in England for people who are homeless. 2018;105–105. Reference Source

[33] Pleace N Filipović Hrast M Benjaminsen L: The Costs of Homelessness in Europe: An Assessment of the Current Evidence Base. Brussels: FEANTSA; (European Observatory on Homelessness Comparative Studies on Homelessness).2013 Reference Source

[34] The King’s Fund: Delivering health and care for people who sleep rough: Going above and beyond.London;2020 Reference Source

[35] Department of Housing, Planning & Local Government: Homelessness Report May 2020.Dublin;2020 Reference Source

[36] Department of the Environment, Community and Local Government: Independent Review of Homeless Services. 2015 Reference Source

[37] The Faculty for Homeless and Inclusion Health: Homeless and Inclusion Health standards for commissioners and service providers.London;2018;64 Reference Source

[38] Gill P MacLeod U Lester H: Improving access to health care for Gypsies and Travellers, homeless people and sex workers. 2013;42 Reference Source

[39] Page A Hilbery O: Turning the tide: a vision paper for multiple needs and exclusions. London;2011 Reference Source

[40] Cortis N Blaxland M: Workforce Issues in Specialist Homelessness Services.Social Policy Research Centre, UNSW Sydney, Sydney;2017[cited 2020 Aug 24]. Reference Source

[41] Mahon E: More Than a Caring Personality: Factors Affecting Staff Retention in Non Profit Organisations in Ireland? *Ir Soc Work.* 2016;8 Reference Source

[42] The Queen’s Nursing Institute: Nursing care for people experiencing homelessness: A Survey of the QNI Homeless Health Network.London: The Queen’s Nursing Institute;2018[cited 2020 Sep 9];20 Reference Source

[43] Focus Ireland: Recommendations to Government: Budget 2020.2019 Reference Source

[44] Cabinet Office Social Exclusion Task Force: Inclusion health: Improving the way we meet the primary health care needs of the socially excluded.London;2010 Reference Source

[45] Making Every Adult Matter Coalition: Solutions from the Frontline.London;2015 Reference Source

[46] Homeless Link and St Mungo’s: Improving hospital admission and discharge for people who are homeless. 2012 Reference Source

[47] Siersbaek R: How accessible is healthcare for single adults experiencing long-term homelessness and complex needs? A realist evaluation protocol.2020 10.17605/OSF.IO/GCHFD PMC7836031 33537554

[48] Farmer T Robinson K Elliott SJ: Developing and Implementing a Triangulation Protocol for Qualitative Health Research. *Qual Health Res.* 2006;16(3):377–94. 10.1177/1049732305285708 16449687

