# Supplementary material for: Making healthcare accessible for single adults with complex needs experiencing long-term homelessness: A realist evaluation protocol
Source: HRB Open Res. 2021 Jan 21;3:73. Originally published 2020 Oct 2. [Version 2] doi: 10.12688/hrbopenres.13154.2 (PMC7836031; doi:10.12688/hrbopenres.13154.2)
Supplement: Peer review history

# Peer review: Reviewer response for version 2

**DOI:** 10.21956/hrbopenres.14355.r28712

**Published:** 2021-02-08

**Authors:** Nigel Hewett

**Affiliations:** 1 Pathway Charity, London, UK

No new comments.

Is the study design appropriate for the research question?

Yes

Is the rationale for, and objectives of, the study clearly described?

Yes

Are sufficient details of the methods provided to allow replication by others?

Yes

Are the datasets clearly presented in a useable and accessible format?

Yes

Reviewer Expertise:

Inclusion health.

I confirm that I have read this submission and believe that I have an appropriate level of expertise to confirm that it is of an acceptable scientific standard.

# Peer review: Reviewer response for version 2

**DOI:** 10.21956/hrbopenres.14355.r28713

**Published:** 2021-01-25

**Authors:** Barbara Clyne

**Affiliations:** 1 Royal College of Surgeons in Ireland, Dublin, Ireland

The authors have addressed my comments. The research findings have great potential for practical use in future service planning and delivery. I look forward to reading future papers.

Is the study design appropriate for the research question?

Yes

Is the rationale for, and objectives of, the study clearly described?

Yes

Are sufficient details of the methods provided to allow replication by others?

Partly

Are the datasets clearly presented in a useable and accessible format?

Not applicable

Reviewer Expertise:

Evidence synthsis, designing and conducting RCTs, process evaluations.

I confirm that I have read this submission and believe that I have an appropriate level of expertise to confirm that it is of an acceptable scientific standard.

# Response

**Published:** 2021-01-25

**Authors:** Rikke Siersbaek

**Affiliations:** Trinity College Dublin, Ireland

Thanks very much for your helpful feedback!

# Peer review: Reviewer response for version 1

**DOI:** 10.21956/hrbopenres.14273.r28276

**Published:** 2020-11-24

**Authors:** Barbara Clyne

**Affiliations:** 1 Royal College of Surgeons in Ireland, Dublin, Ireland

This paper describes a protocol for a realist evaluation of funding procedures and health system performance management on accessibility of services for adults experiencing chronic homelessness. Research on this vulnerable population represents an important area of health services research and the findings could have important policy impacts. The research builds on a previous realist review by the study team, demonstrating their expertise in this area.

 The protocol is clear, well written and the overall study is well described. However, there are a number of clarifications that I feel would improve the manuscript overall. These are as follows:

- Title: The title and the study aim don’t seem to align to me. The title “How accessible is healthcare for single adults experiencing long-term homelessness and complex needs?” implies that this study is about accessing healthcare from the perspective of adults experiencing homelessness. But the study aim is to understand how funding procedures and health system performance management impact service settings, staff, providers and their ability to make services accessible. So providers of homeless services and the system are really the focus of the study. If providers can make the service available, is different to whether or not adults experiencing homelessness then access it. I think this is especially the case for the focus group with adults who previously experienced homelessness who may or may not be part of the study. There is also a little contradiction within the introduction, 4 ^th^ paragraph, the focus of this research is homeless populations, and 5 ^th^ paragraph, aim to understand system perspective. Suggestion: making healthcare accessible for homeless adults....
- The introduction gives a good justification as to why realist evaluation is suited to exploring barriers to health care access from the perspective of homeless populations, but as above, the focus of this study is the system so a sentence or two justifying why this approach is suitable would be good.

- The research builds on a previous realist review by the study team. Figure 2 outlining the full programme theory is a nice visual, but I think a full statement of what the programme theory is would be beneficial.
- The protocol is focused on 2 of the 6 elements of the overall theory from the realist review. Could the authors provide brief info on the other 4 elements and a more detailed description of why these 2 elements were selected over the other 4. It would also be helpful if the authors reflected on how the focus on 2 elements only impacts the overall theory?
- For consistency, it would be helpful to describe CMOC 2 consistently with the rest of the manuscript in terms of calling it performance management, as opposed to goals and incentives. Or define what is meant by performance management earlier on.
- Realist evaluations tend to require good data on outcomes and where possible, data about outcomes should be triangulated (at least using different sources, if not different types, of information). In terms of your CMOCs, where is the outcome data coming from? In terms of data collection overall, I think it would be helpful to provide a table/figure demonstrating the data source for each of the CMOs to ensure you have all the adequate data.
- For your realist interview approach, you will be interviewing key stakeholders. With this approach, they will be asked to contradict/refine your theory. Could you comment on how you might reduce social desirability bias and get the respondents to really challenge the researcher. How experienced in their area will the participants be and how senior/junior will the researcher be?
- Will you be using any reporting standards such as RAMESES II?

Is the study design appropriate for the research question?

Yes

Is the rationale for, and objectives of, the study clearly described?

Yes

Are sufficient details of the methods provided to allow replication by others?

Partly

Are the datasets clearly presented in a useable and accessible format?

Not applicable

Reviewer Expertise:

Evidence synthsis, designing and conducting RCTs, process evaluations.

I confirm that I have read this submission and believe that I have an appropriate level of expertise to confirm that it is of an acceptable scientific standard, however I have significant reservations, as outlined above.

# Response

**Published:** 2020-12-19

**Authors:** Rikke Siersbaek

**Affiliations:** Trinity College Dublin, Ireland

(Comments in bold – answers in normal)

 18 Dec 2020

**This paper describes a protocol for a realist evaluation of funding procedures and health system performance management on accessibility of services for adults experiencing chronic homelessness. Research on this vulnerable population represents an important area of health services research and the findings could have important policy impacts. The research builds on a previous realist review by the study team, demonstrating their expertise in this area.**

**The protocol is clear, well written and the overall study is well described. However, there are a number of clarifications that I feel would improve the manuscript overall. These are as follows:**

**Title: The title and the study aim don’t seem to align to me. The title “How accessible is healthcare for single adults experiencing long-term homelessness and complex needs?” implies that this study is about accessing healthcare from the perspective of adults experiencing homelessness. But the study aim is to understand how funding procedures and health system performance management impact service settings, staff, providers and their ability to make services accessible. So providers of homeless services and the system are really the focus of the study. If providers can make the service available, is different to whether or not adults experiencing homelessness then access it. I think this is especially the case for the focus group with adults who previously experienced homelessness who may or may not be part of the study. There is also a little contradiction within the introduction, 4th paragraph, the focus of this research is homeless populations, and 5th paragraph, aim to understand system perspective. Suggestion: making healthcare accessible for homeless adults....**

 Yes good point. Thank you for that suggestion. We have amended the title.

**The introduction gives a good justification as to why realist evaluation is suited to exploring barriers to health care access from the perspective of homeless populations, but as above, the focus of this study is the system so a sentence or two justifying why this approach is suitable would be good.**

 Thank you for pointing out the need to have more detail here. We have added more text to the document.

**The research builds on a previous realist review by the study team. Figure 2 outlining the full programme theory is a nice visual, but I think a full statement of what the programme theory is would be beneficial.**

 Might you be referring to Figure 1 which is the full programme theory? We do briefly explain the full programme theory in the paragraph just before figure 1 but we have added more content to provide a fuller explanation.

**The protocol is focused on 2 of the 6 elements of the overall theory from the realist review. Could the authors provide brief info on the other 4 elements and a more detailed description of why these 2 elements were selected over the other 4. It would also be helpful if the authors reflected on how the focus on 2 elements only impacts the overall theory?**

 Thank you for this suggestion.

 We have chosen not to describe each of the six CMOCs in the protocol because they are included in the full set of findings described in the full programme theory and because to do so it would repeat a lot of the realist review in which they are each fully explained and which is available as a preprint (https://www.researchsquare.com/article/rs-79236/v1) (and hopefully soon will be published!) We selected the two which, based on the existing literature, had the greatest potential for impact while also being under-researched and we felt that two provide the right balance of depth and breadth. We are not able to comprehensively build on each of the CMOCs from the review and the two were chosen as a starting point for the realist evaluation because as they are under-researched and policy relevant. It is important to note also that they are just a starting point which will guide the initial data collection and initial coding of data in the evaluation. The end product will move beyond them to produce a number of subsequent CMOCs which will be refined and combined to produce a set of CMOCs and a final programme theory for the realist evaluation.

**For consistency, it would be helpful to describe CMOC 2 consistently with the rest of the manuscript in terms of calling it performance management, as opposed to goals and incentives. Or define what is meant by performance management earlier on.**

 Good point. Have made that change in the graphic and replaced and have updated text as well.

**Realist evaluations tend to require good data on outcomes and where possible, data about outcomes should be triangulated (at least using different sources, if not different types, of information). In terms of your CMOCs, where is the outcome data coming from? In terms of data collection overall, I think it would be helpful to provide a table/figure demonstrating the data source for each of the CMOs to ensure you have all the adequate data.**

 The two CMOCs carried over will be a starting point for generating many more using the data collected for this study. New CMOCs will be developed to further investigate the topics explored in these two CMOCs in further detail. To this end, the data for the study, including outcomes data, will be generated from policy documents, interviews and focus groups. Additionally, we’ll use other data sources such as funding information from HSE Social Inclusion to understand how funding flows to service providers in the voluntary sector, as well as peer reviewed and grey literature which discuss the topics. Each CMOC will always draw on several data sources. At this stage, since the CMOCs have not been constructed we cannot include a table to demonstrate the data source for each of them as that will depend on the data collection to come.

**For your realist interview approach, you will be interviewing key stakeholders. With this approach, they will be asked to contradict/refine your theory. Could you comment on how you might reduce social desirability bias and get the respondents to really challenge the researcher. How experienced in their area will the participants be and how senior/junior will the researcher be?**

 The interview technique is informed by a number of sources (eg (1,2)) as well as practical learning undertaken by the primary researcher in trainings provided by experts in the field (Geoff Wong and Justin Jagosh). As outlined in the RAMESE guidelines for realist interviews(1), theory testing in the interview setting is a process that takes place bit by bit and the researcher’s theories are typically not introduced in their full form nor formally as ‘here is my theory.’:

 ‘Interviews should start with general questions about interviewees’ role in, experiences of and views about the programme. Subsequent questions follow up their responses, asking them to tell their stories about specific experiences or issues with the programme, its participants, and constraints. Each of these questions investigates something about the programme theory being tested. Specific elements of programme theory can then be explicitly introduced and tested with the respondent. In order to avoid allegations of ‘leading the interview’, it is a good idea to test multiple, including contradictory, theories about the same aspect of the programme with the same respondent’. (1)

 The realist interview process is often described as a ‘teacher-learner’ process whereby the interviewer presents theories under examination using normal (non-realist) language as the ‘teacher’ explaining some aspect of their findings to the interviewee. For example, in our study the researcher may ask ‘I have heard from other people I have spoken with (or I have read in the published literature) that funding cycles in health services from homeless populations are often short and unstable. Is that your experience? Could you tell me more about that?’ to begin to test CMOC1. The teacher and learner roles are not static – the interviewer moves fluidly back and forth. We may then, moving into learner mode, depending on what the interviewee answers, follow up asking about the effects of long or short or mixed funding cycles they’ve experienced in relation to staffing stability and their own experience of working for a given service. We might then ask about other aspects of the topic such as, for example explaining that in the literature we have read that that people working at the frontline are seen by their managers as naturally caring people who are rewarded by doing caring work and therefore are not always compensated fairly, and get their thoughts on that. And so on and so forth. (1,2)

 In this manner, the causal relationship between mechanisms and outcomes is investigated by asking about specific aspects of the theory rather than by laying out the whole theory and asking for validation. This should diminish the risk of social desirability bias as the researcher does not present theories as ‘my work’ but rather as what they’ve been told by other interviewees or learned from the literature, and often will present contradictory findings and points of view for discussion with the interviewee. 

 Interviewees will be frontline medics, nurses and social workers and service managers with many years’ experience as well one individual from HSE Social Inclusion (programme management level). Others will likely be added per snowballing.

 The researcher undertaking the interviews is a PhD student. 

**Will you be using any reporting standards such as RAMESES II?**

 We will not formally report on each of the aspects of the RAMESES II checklist but it will be used to guide and to continually check the quality of our work throughout the process.

**References**

 1.            Greenhalgh T, Pawson G, Westhorp G, Greenhalgh J, Manzano A, Jagosh J. The Realist Interview: The RAMESES II Project. 2017;1–3.

 2.            Manzano A. The craft of interviewing in realist evaluation. Evaluation. 2016 Jul 1;22(3):342–60.

# Peer review: Reviewer response for version 1

**DOI:** 10.21956/hrbopenres.14273.r28256

**Published:** 2020-11-18

**Authors:** Nigel Hewett

**Affiliations:** 1 Pathway Charity, London, UK

This paper sets out a protocol for a realist evaluation approach to understanding the the impact of funding procedures and health system performance management on accessibility of services for people experiencing homelessness. 

 The protocol is clearly explained and the study design is practical and takes account of potential difficulties related to the COVID pandemic. 

 The realist evaluation approach is of increasing interest in the field of inclusion health, and this protocol looks likely to result in research findings that are of real practical use for future service planning and delivery.

Is the study design appropriate for the research question?

Yes

Is the rationale for, and objectives of, the study clearly described?

Yes

Are sufficient details of the methods provided to allow replication by others?

Yes

Are the datasets clearly presented in a useable and accessible format?

Yes

Reviewer Expertise:

Inclusion health.

I confirm that I have read this submission and believe that I have an appropriate level of expertise to confirm that it is of an acceptable scientific standard.

# Response

**Published:** 2020-12-19

**Authors:** Rikke Siersbaek

**Affiliations:** Trinity College Dublin, Ireland

Thank you very much for your feedback. Much appreciated!
